# In-depth plasma proteomics reveals increase in circulating PD-1 during anti-PD-1 immunotherapy in patients with metastatic cutaneous melanoma

**DOI:** 10.1136/jitc-2019-000204

**Published:** 2020-05-20

**Authors:** Haris Babačić, Janne Lehtiö, Yago Pico de Coaña, Maria Pernemalm, Hanna Eriksson

**Affiliations:** 1 Department of Oncology and Pathology, Karolinska Institute, Stockholm, Sweden; 2 Theme Cancer/Department of Oncology/Skin Cancer Centre, Karolinska University Hospital, Stockholm, Sweden

**Keywords:** programmed cell death 1 receptor, immunotherapy, melanoma, tumor biomarkers

## Abstract

**Background:**

Immune checkpoint inhibitors (ICIs) have significantly improved the outcome in metastatic cutaneous melanoma (CM). However, therapy response is limited to subgroups of patients and clinically useful predictive biomarkers are lacking.

**Methods:**

To discover treatment-related systemic changes in plasma and potential biomarkers associated with treatment outcome, we analyzed serial plasma samples from 24 patients with metastatic CM, collected before and during ICI treatment, with mass-spectrometry-based global proteomics (high-resolution isoelectric focusing liquid chromatography–mass spectrometry (HiRIEF LC-MS/MS)) and targeted proteomics with proximity extension assays (PEAs). In addition, we analyzed plasma proteomes of 24 patients with metastatic CM treated with mitogen-activated protein kinase inhibitors (MAPKis), to pinpoint changes in protein plasma levels specific to the ICI treatment. To detect plasma proteins associated with treatment response, we performed stratified analyses in anti-programmed cell death protein 1 (anti-PD-1) responders and non-responders. In addition, we analyzed the association between protein plasma levels and progression-free survival (PFS) by Cox proportional hazards models.

**Results:**

Unbiased HiRIEF LC-MS/MS-based proteomics showed plasma levels’ alterations related to anti-PD-1 treatment in 80 out of 1160 quantified proteins. Circulating PD-1 had the highest increase during anti-PD-1 treatment (log2-FC=2.03, p=0.0008) and in anti-PD-1 responders (log2-FC=2.09, p=0.005), but did not change in the MAPKis cohort. Targeted, antibody-based proteomics by PEA confirmed this observation. Anti-PD-1 responders had an increase in plasma proteins involved in T-cell response, neutrophil degranulation, inflammation, cell adhesion, and immune suppression. Furthermore, we discovered new associations between plasma proteins (eg, interleukin 6, interleukin 10, proline-rich acidic protein 1, desmocollin 3, C-C motif chemokine ligands 2, 3 and 4, vascular endothelial growth factor A) and PFS, which may serve as predictive biomarkers.

**Conclusions:**

We detected an increase in circulating PD-1 during anti-PD-1 treatment, as well as diverse immune plasma proteomic signatures in anti-PD-1 responders. This study demonstrates the potential of plasma proteomics as a liquid biopsy method and in discovery of putative predictive biomarkers for anti-PD-1 treatment in metastatic CM.

## Background

Novel therapies with immune checkpoint inhibitors (ICIs), that is, anti-CTLA-4 and anti-programmed cell death protein 1 (anti-PD-1) ICIs, have dramatically improved the outcomes for patients with metastatic cutaneous melanoma (CM).^1–5^ According to long-term follow-up of phase III clinical trials, the PD-1 inhibitors (ie, pembrolizumab and nivolumab) have shown superiority compared with the cytotoxic T-lymphocyte–associated antigen 4 (CTLA-4) inhibitor ipilimumab.^2 4^ Among patients receiving ICIs as first-line therapy, the median overall survival (OS) was 38.7 months for pembrolizumab versus 17.1 months for ipilimumab (HR=0.73, p=0.0036) with survival rates at 5 years of 43.2% and 33.0%, respectively.^4^ In patients with ICIs as a second-line treatment, the median OS was 23.5 months for pembrolizumab versus 13.6 months for ipilimumab (HR=0.75, p=0.036). A therapeutic combination of anti-PD-1 and anti-CTLA-4 inhibitors has further improved the response rates and OS.^5^ Objective response rates were 58% for nivolumab plus ipilimumab, 45% for nivolumab alone, and 19% for ipilimumab alone. This also reflected in increasing the 5-year OS rates up to 52% for the nivolumab plus ipilimumab combination, as compared with 44% for nivolumab monotherapy and 26% for ipilimumab monotherapy. Aside from ICIs, patients with a BRAFv600-mutated metastatic CM have another therapeutic option in targeted therapy with mitogen-activated protein kinase inhibitors (MAPKis), that is, BRAF inhibitors and MEK inhibitors (BRAFis/MEKis), which in combination have extended the 5-year OS up to 34%.^6^

Still, a proportion of patients with metastatic CM are non-responders or develops resistance to ICI treatment, with approximately 40% not responding even to the combination of anti-PD-1 and anti-CTLA-4.^3^ Although there is a need to predict the response to treatment, to date, there are no validated biomarkers in clinical practice for predicting anti-PD-1 treatment outcome in metastatic CM. Tumor mutational burden, tumor infiltrating lymphocytes, gene and protein expression in tissue, neutrophil to lymphocyte ratio (NLR), derived NLR, PD-L1 expression on the tumor cells and the gut microbiota are all examples of biomarkers for ICIs that have been investigated in CM.^7–11^ Moreover, growing evidence obtained by liquid biopsy approaches has shown that the profound heterogeneity between different subclones in metastatic CM disease has an effect on response to therapy and development of resistance to treatment.^12 13^ Beyond the ongoing research in the field, no studies to detect systemic changes related to ICIs treatment have been performed using state-of-the-art global proteomics methods that can provide wide, unbiased identification of the plasma proteome.

Recently, we have shown a robust detection and quantification of over 1000 proteins in human plasma, including tissue leakage proteins and cancer signaling proteins by mass spectrometry-based proteomics (HiRIEF LC-MS/MS).^14^ This method provides us with a unique and previously unexplored view into the dynamic changes of the proteins present in plasma. To study the ongoing systemic biological processes as well as circulating and tumor-derived proteins associated with treatment outcome, we have combined global and targeted proteomic methods, using both unbiased global proteomics (high-resolution isoelectric focusing liquid chromatography–mass spectrometry (HiRIEF LC-MS/MS))^14 15^ and antibody-based, targeted proteomics with proximity extension assays (PEAs)^16^ in prospectively collected, sequential plasma samples from patients with metastatic (stage IV) CM treated with ICIs. To detect potential plasma biomarkers, we analyzed the change in the plasma proteome attributable to ICI treatment and estimated the predictive relation between protein plasma levels and progression-free survival (PFS). In parallel, we analyzed a cohort of patients receiving targeted therapy with MAPKis, to differentiate effects specific to ICI treatment from other response markers.

## Methods

**Figure 1:**
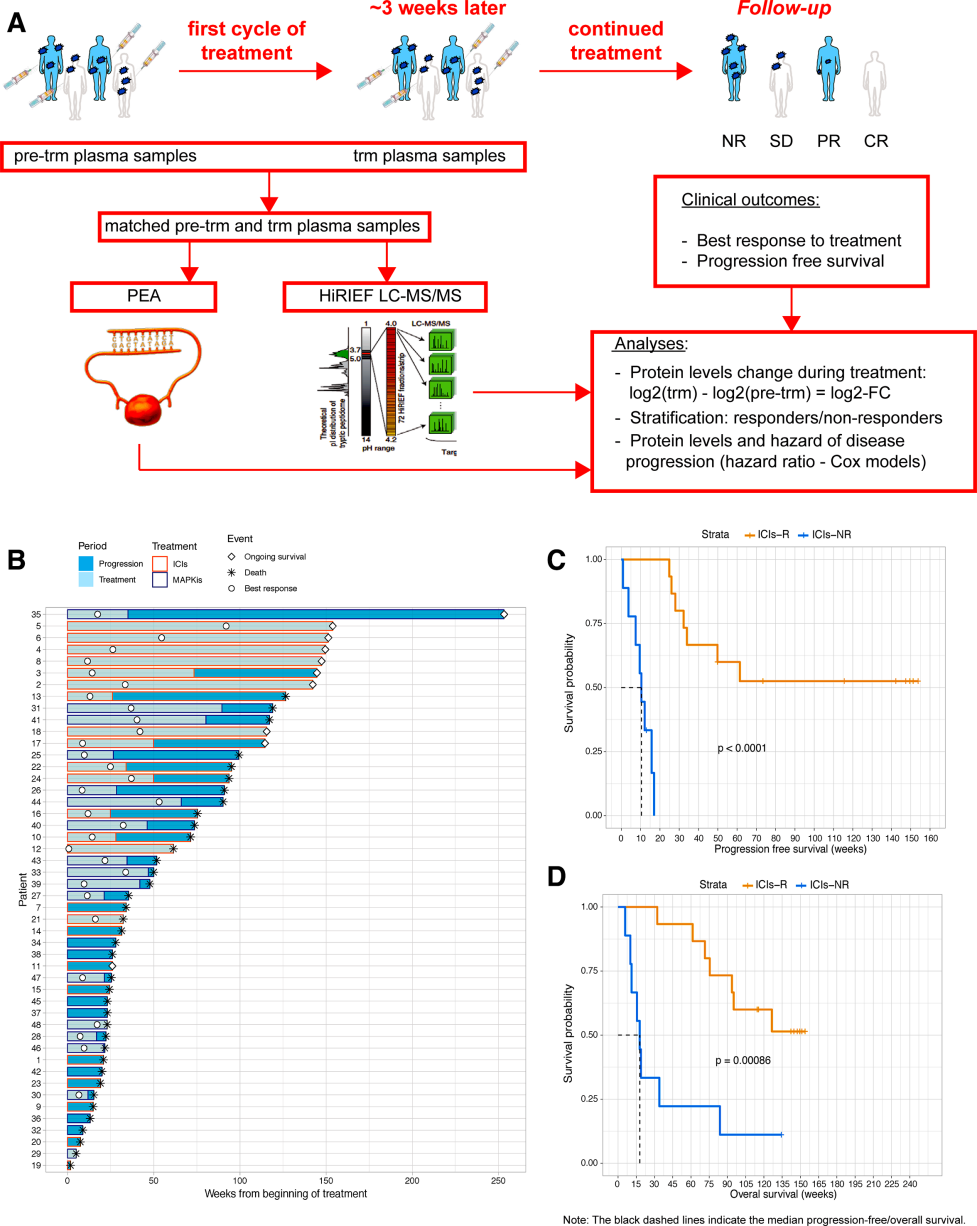
Sequential biobanking of plasma samples and treatment outcome of all patients included in the study. (A) Workflow of the study. Plasma samples were collected pretreatment at baseline and during treatment with ICIs in the study cohort or with MAPKis in the comparison cohort. Treatment outcomes were followed prospectively. (B) Swimmers’ plot on patient and treatment follow-up. All patients, except patients 35, 6, 4, and 3, had matched samples for PEA analyses. Patient 35 received MAPKis as first-line treatment, followed by ICIs at progression and is the longest survivor—this is the most evident example how additional treatments after progression can affect overall survival and why analyzing progression-free survival is a more valid outcome in this study.; (C and D) Kaplan-Meier curves on progression-free survival (C) and overall survival (D) in patients on ICIs, stratified per treatment response (p values—two-sided log-rank test). PEA image courtesy of Olink Proteomics AB. CR, complete response; HiRIEF LC-MS/MS, high-resolution isoelectric focusing liquid chromatography–mass spectrometry; ICIs, immune checkpoint inhibitors; ICIs-NR, patients treated with ICIs with no response to treatment; ICIs-R, patients treated with ICIs with response to treatment; log2-FC, log2-fold-change; MAPKis, mitogen-activated protein kinase inhibitors; NR, no response; PEA, proximity extension assays; PR, partial response; pre-trm, pre-treatment; SD, stable disease; trm, after the first treatment cycle.

The research design is described in figure 1A. Details on methods are available in online supplementary additional file 1. Brief description is provided below.

10.1136/jitc-2019-000204.supp1 Supplementary data

### Patient characteristics and plasma samples

Serial plasma samples were collected before treatment (pre-trm) and after the first treatment cycle (trm), before the second cycle, from 46 patients with metastatic (stage IV) CM treated with first-line ICIs (ie, anti-CTLA-4 and/or anti-PD-1) or MAPKis (MEKis and/or BRAFis). The blood samples were centrifuged and the plasma was stored at −70°C until further analysis.

The clinical data included age at treatment start, sex, baseline M-stage according to American Joint Committee on Cancer, Eighth Edition^17^ (M1a, M1b, M1c-d), baseline lactate dehydrogenase (LDH) levels, best response to treatment, date of disease progression, and/or date of death.^18^

Best response to treatment was based on clinical and/or radiological investigations (ie, CT, MRI and/or positron emission CT tomography), evaluated by oncologists and radiologists. Responders were defined as patients with complete response (CR) or stable disease (SD) or partial response (PR) (ie, decreased number and/or size of the existing metastases), without appearance of new lesions confirmed by imaging and/or clinical examination. Non-responders were defined as patients with lesions of increasing size or new lesions shown by imaging and/or clinical examination without any previous response to the therapy. Date of progression was recorded at the time of the confirmatory imaging. The PFS was defined as the time from treatment start until the date of confirmed progression or date of death. The OS was defined as the time from treatment start until date of death.

All patients but four were treated outside of clinical trials and were followed according to the standard clinical follow-up scheduled every fourth week, with radiological evaluation performed every 8–12 weeks. One patient received treatment within COLUMBUS/NCT01909453, one in COMBI-d/NCT01584648, and two in CheckMate 401/NCT02599402. The clinical trials have been reported. The patients within clinical trials were per protocol and were evaluated with imaging according to RECIST 1.1 (Response Evaluation Criteria in Solid Tumors, Version 1.1).^19^

### Mass-spectrometry-based proteomics analysis

#### High-abundant protein depletion and in-solution digestion

The most abundant proteins in plasma were depleted with the Agilent Plasma 14 Multiple Removal System 4.6×100, which was set up on an Agilent HPLC system (Agilent Technologies). Protein concentration was determined by Bio-Rad DC Assay and equal amounts of each sample was subjected to in-solution digestions with LysC and trypsin.

#### Tandem mass tags labeling

Before labeling, equal amounts of peptide samples were pH adjusted using triethylammonium bicarbonate buffer, pH 8.5. The resulting peptide mixtures were labeled with isobaric tandem masstags (Thermo Scientific). Labeling efficiency was determined by liquid chromatography–massspectrometry (LC-MS/MS) before pooling of samples. Subsequently, sample clean-up was performed by solid phase extraction (SPE strata-X-C, Phenomenex). The labeling scheme can be found in online supplementary figure S1 additional file 1.

#### High-resolution isoelectric focusing with LC-MS/MS

The HiRIEF prefractionation method at peptide level was applied as previously described.^15^ Briefly, after sample clean-up by solid phase extraction (SPE strata-X-C, Phenomenex), the sample pool was subjected to peptide IEF-IPG (isoelectric focusing by immobilized pH gradient) in pI range 3–10 (1 mg). The freeze-dried peptide sample was dissolved in 250 µL rehydration solution containing 8M urea and allowed to adsorb to the gel strip by swelling overnight. The 24 cm linear gradient IPG strip (GE Healthcare) was incubated overnight in 8M rehydration solution containing 1% IPG pharmalyte pH 3–10 (GE Healthcare). After focusing, the peptides were passively eluted into 72 contiguous fractions with MilliQ water/35% acetonitrile/35% acetonitrile and 0.1% formic acid, using an in-house constructed IPG extractor robotics (GE Healthcare Biosciences AB, prototype instrument) into a 96-well plate (V-bottom, Greiner product no 651201). The resulting fractions were then freeze-dried and kept at −20°C until LC-MS/MS analysis and data searches (see online supplementary additional file 1).

#### Proteogenomics search pipeline/SpectrumAI

The HiRIEF LC-MS/MS data were searched in a previously described customized peptide database named varDB6 (15). The detected novel peptides were then curated using the SpectrumAI pipeline, described elsewhere.^20^ We then removed 11 peptides with n>D, D<N or Q>E, E<Q substitutions since the low mass difference between these amino acids increases the risk of co-isolation of isotopic variants in the HiRIEF LC-MS/MS analysis and generation of false positives.

### Proximity extension assays

The plasma samples were additionally analyzed using PEAs at the clinical biomarkers’ facility at SciLifeLab, Uppsala, Sweden. In total, 92 human protein biomarkers (online supplementary table S0, additional file 2) were measured using Olink ImmunoOnc I panel (www.olink.com).^16^ The final assay readout is presented in normalized protein expression values, which is an arbitrary unit on a log2-scale where a higher value corresponds to a higher protein expression.

10.1136/jitc-2019-000204.supp2 Supplementary data

### Statistical analysis

We included only anti-PD-1-treated patients and all MAPKi-treated patients in the analyses (online supplementary figure S1a, additional file 1), including only proteins detected in more than 50% and 80% of the observations in HiRIEF and PEA data, respectively. HiRIEF and PEA analyses were performed separately. All proteins were annotated with corresponding gene names. The protein quantifications in both HiRIEF LC-MS/MS and PEA data were log2-normalized. To analyze the change of protein levels in the plasma during treatment, we compared protein levels in trm plasma samples matched with the corresponding pre-trm samples, using a paired two-sided t test, at α=0.05.

To further investigate changes specific to response to anti-PD-1 treatment, we stratified anti-PD-1-treated patients into subgroups of anti-PD-1 responders (anti-PD-1-R) and anti-PD-1 non-responders (anti-PD-1-NR). Plasma proteomes between trm and pre-trm matched samples were compared in the separate strata of anti-PD-1-R and anti-PD-1-NR, using a paired two-sided t test. The change in plasma levels of each protein during treatment was quantified with the log2-fold-change (log2-FC). Furthermore, we compared the log2-FC in anti-PD-1-R with anti-PD-1-NR with a two-sided unpaired t test. To address uncertainty due to response categorization, we performed sensitivity analyses using only patients with CR (anti-PD-1-CR) as representative of responders.

We analyzed the association between protein plasma levels and PFS using Cox proportional hazards models, performing univariate and multivariate analyses (adjusting for clinical variables that were associated with PFS in the corresponding subgroup). Furthermore, in sensitivity multivariate analyses, we adjusted the Cox models for age, sex, and LDH levels (>normal vs normal), regardless of whether these clinical variables were initially associated with PFS.

Extensive details on agreement analysis, multiple testing, stratification, sensitivity analyses, and Cox models are available in online supplementary information additional file 1. All analyses were performed in R, V.3.5.1.

## Results

### Patients’ cohorts and treatment

The analyzed samples were taken from 24 patients with metastatic CM undergoing first-line ICI treatment and 24 patients with metastatic CM receiving MAPKi treatment (figure 1B; online supplementary table S1, additional file 2). Responders to ICI treatment (ICI-R), that is, patients with CR, PR, or SD, and non-responders to ICI treatment (ICI-NR) did not have differences in clinical characteristics at baseline (table 1). ICI-R had longer PFS and OS compared with ICI-NR (figure 1C and D).

**Table 1:** Patient and clinical characteristics of patients with metastatic cutaneous melanoma receiving ICIs

Patients and clinical characteristics	ICIs-R (n=15)	ICIs-NR (n=9)	P value
Median age at treatment start (years)	72±15.81	70±12.28	0.440
Females, n (%)	9 (60.00)	2 (22.22)	0.105
Males, n (%)	6 (40.00)	7 (77.78)	
Baseline M-stage, n (%)			
M1a	1 (6.67)	0	1
M1b	4 (26.67)	2 (22.22)	
M1c-d	10 (66.67)	7 (77.78)	
Baseline LDH			
Median (μkat/L)	3.95±1.11	3.90±7.36	0.850
≤ULN, n (%)	8 (53.33)	4 (44.44)	0.680
>ULN, n (%)	6 (40.00)	5 (55.56)	
Missing data, n (%)	1 (6.67)	0	
First-line therapy, n (%)			
Anti-PD-1	10 (66.67)	6 (66.67)	0.551
Anti-CTLA-4	3 (20.00)	3 (33.33)	
Anti-PD-1 and anti-CTLA-4	2 (13.33)	0	
Best treatment response, n (%)			
Complete response	5 (33.33)	0	n.a
Partial response	4 (26.67)	0	
Stable disease	6 (40.00)	0	
Progressive disease (no response)	0	9 (100.00)	
Progression-free survival, median (days)	430±375.20	73±37.15	<0.001
Overall survival, median (days)	809±269.12	125±305.24	<0.001

P-values are obtained with t, Wilcoxon, Fisher, or log-rank test, number after ± is SD.

ICIs-NR, patients treated with immune checkpoint inhibitors with no response to treatment; ICIs-R, patients treated with immune checkpoint inhibitors with response to treatment; n.a., not applicable.

### Plasma samples and proteomes

To detect changes in the plasma proteome attributable to treatment, we analyzed serial pre-trm and trm plasma samples with a median of 20 days between samples (range: 7–57, SD: 10.26).

We used two complementary methods, HiRIEF LC-MS/MS and PEA, to ensure a comprehensive overview of plasma proteins. HiRIEF LC-MS/MS provides an unbiased and wide coverage of the plasma proteome,^14^ with the potential to detect proteins with sequence variants, whereas PEAs quantify selected proteins of interest.^16^ In total, we analyzed 45 plasma samples from 24 patients with HiRIEF LC-MS/MS (21 matched), and 86 plasma samples from an extended cohort of 45 patients using PEA assays (41 matched). The MAPKi-treated patients were included to provide a comparison group for proteome changes not specific to anti-PD-1 treatment. This follows the assumption that changes attributable to anti-PD-1 treatment would not have appeared in patients treated with MAPKis (online supplementary figure S1, additional file 1).

Overall, HiRIEF LC-MS/MS detected 1835 unique proteins. Of these, 1160 proteins had at least 50% of observations, and 77 out of 92 immuno-oncology-related proteins had at least 80% observations in PEA data (online supplementary figure S2, additional file 1). HiRIEF LC-MS/MS detected 10 of the 92 PEA proteins (10.87%): PDCD1 (ie, PD-1), programmed cell death 1 ligand 2 (PDCD1LG2/PD-L2), tumor necrosis factor receptor superfamily member 21 (TNFRSF21), colony stimulating factor 1 (CSF1), ttransforming growth factor beta 1 (TGFB1), angiopoietin-1 receptor (TEK), decorin (DCN), galectin 1 (LGALS1), vascular endothelial growth factor receptor 2 (KDR), and inducible T Cell costimulator ligand (ICOSLG) (online supplementary figure S3, additional file 1).

### Change in protein plasma levels is associated with anti-PD-1 treatment

**Figure 2:**
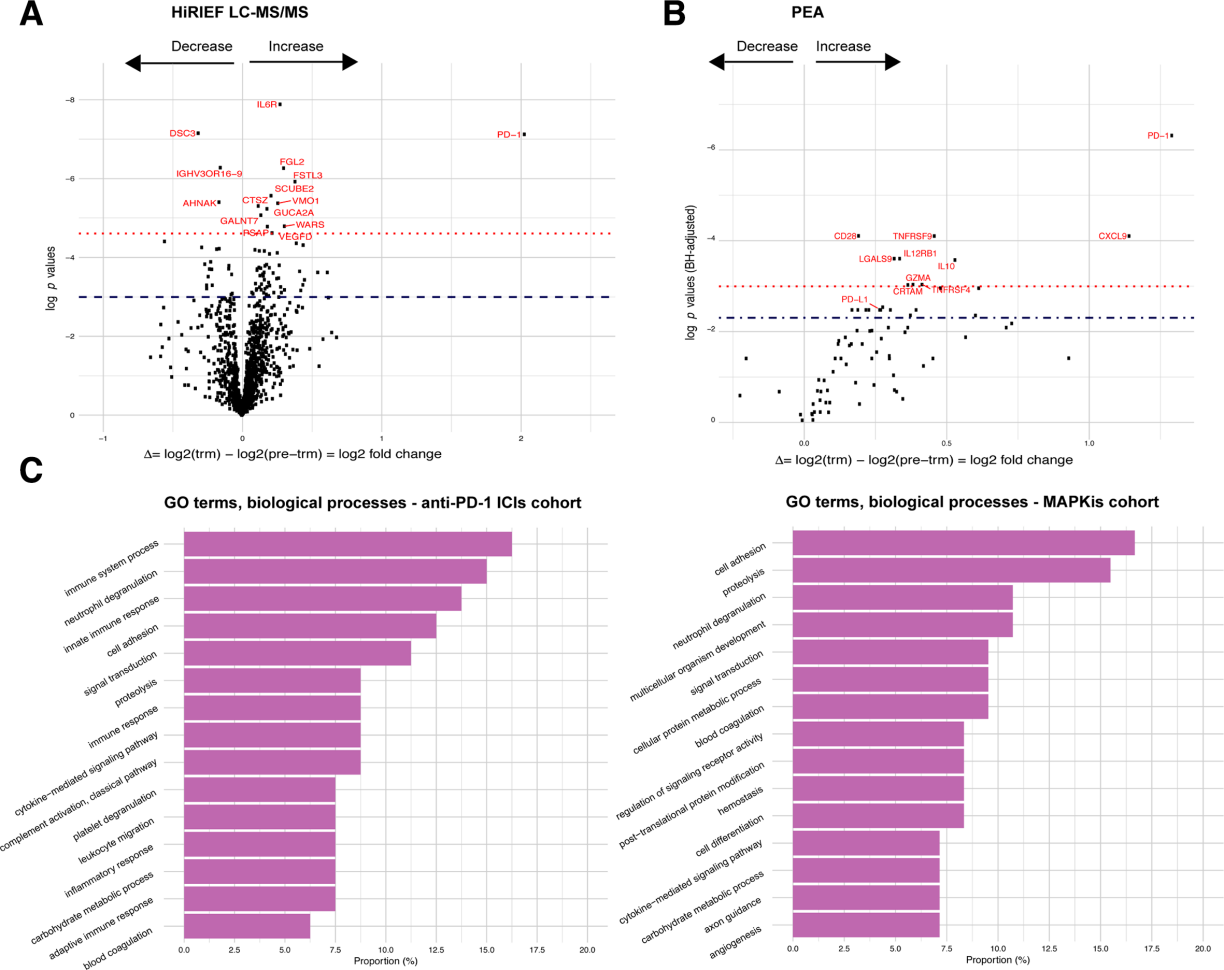
Change (log2-FC) in protein plasma levels during anti-PD-1 treatment in metastatic cutaneous melanoma. (A) Volcano plots on comparing trm with pre-trm protein plasma levels in all patients with matched samples who were treated with anti-PD-1 ICIs, HiRIEF LC-MS/MS analyses (paired t test, two sided). Proteins above the dashed line = p< 0.05, proteins in red and above the dotted line = p< 0.001 (no FDR). (B) Volcano plots on comparing trm with pre-trm protein plasma levels in all patients with matched samples who were treated with anti-PD-1 ICIs, PEA analyses (paired t test, two sided). Proteins above the dashed line = q< 0.1, proteins in red and above the dotted line = q< 0.05 (p<0.05, 10% FDR). (C) Distribution of GO terms on biological processes of proteins that had a significant change in plasma levels during treatment in the anti-PD-1 treatment cohort (left) and in the MAPKi treatment cohort (right). PD-1, programmed cell death protein 1; GO, gene ontology; HiRIEF LC-MS/MS, high-resolution isoelectric focusing liquid chromatography–mass spectrometry; ICIs, immune checkpoint inhibitors; IL10, interleukin10; log2-FC, log2-fold-change; MAPKis, mitogen-activated protein kinase inhibitors; PEA, proximity extension assays; pre-trm, pre-treatment; trm, after the first treatment cycle.

**Figure 3:**
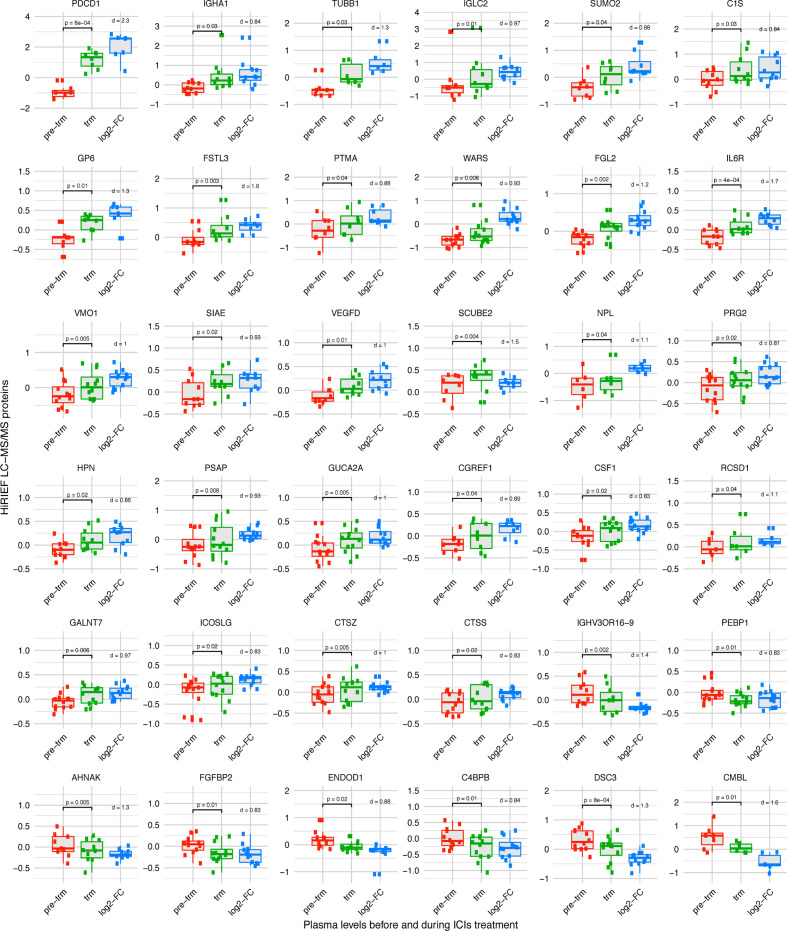
Proteins with a change (log2-FC) in plasma levels during treatment with anti-PD-1 ICIs. Proteins which had the largest effect size (Cohen’s d >0.81) are shown, analyzed with HiRIEF LC-MS/MS (paired t test, two sided, α=0.05). Multiple testing has likely contributed to type I error. However, the discoveries are more reliable in proteins whose change during treatment is of larger effect size (Cohen’s d). Boxplots: center line = median; box limits = upper and lower quartiles; whiskers = 1.5 x IQR; points outside of IQR = outliers. Proteins with log2-FC>0 = increase, log2-FC <0 = decrease; Cohen’s d=0.8 = large effect size, Cohen’s d=1.2 = very large effect size, Cohen’s d=2 = huge effect size. None of these proteins had a change in plasma levels during treatment with MAPKis. PD-1, programmed cell death protein 1; HiRIEF LC-MS/MS, high-resolution isoelectric focusing liquid chromatography–mass spectrometry; ICIs, immune checkpoint inhibitors; IL6R, interleukin-6 receptor; log2-FC, log2-fold-change; MAPKis, mitogen-activated protein kinase inhibitors; pre-trm, pre-treatment; trm, after the first treatment cycle.

First, we set out to explore the overall change in the plasma proteome attributable to anti-PD-1 treatment, regardless of response to treatment. In patients treated with anti-PD-1, 80 proteins had a significant change in plasma levels during treatment in HiRIEF LC-MS/MS data (p<0.05, no false discovery rate (FDR); figure 2A, online supplementary table S2 - additional files 1 and 2), with PD-1 having the largest increase (mean log2-FC=2.03, p<0.001; figure 2A). Two of the 80 proteins (CECR1 and GSS) also showed a change during treatment in the MAPKi cohort. To differentiate which proteins were less likely to be false positives (type I errors) due to multiple testing in HiRIEF data, we calculated the effect size (Cohen’s d) for each of the proteins, assuming that proteins with log2-FC of larger effect sizes were more confident discoveries. The proteins with the largest effect sizes are shown in figure 3.

In the PEA data, 23 out of 77 analyzed proteins had a change in plasma levels during anti-PD-1 treatment (p<0.05, 10% FDR; figure 2B, online supplementary table S3 - additional files 1 and 2). Six of these proteins also had an increase in plasma levels during treatment with MAPKis: CXCL9, interleukin 12, GZMA, CRTAM, galectin 9 (LGALS9), and KLRD1. PD-1 was again observed as having the largest increase during anti-PD-1 treatment (mean log2-FC=1.29, p<0.001, q=0.002), recapitulating the change detected by HiRIEF LC-MS/MS. Interestingly, in the PEA data, programmed cell death 1 ligand 1 (PD-L1) showed an increase during treatment (mean log2-FC=0.267, p=0.022, q=0.084).

To explore the biological functions of the alternating proteins, we analyzed the distribution of gene ontology (GO) terms from the global HiRIEF LC-MS/MS analyses (online supplementary figure S4, additional file 1). The majority of the proteins altered during anti-PD-1 treatment were involved in immune system processes, which was different from the proteins changed due to MAPKi treatment, where most of the proteins were involved in cell adhesion (figure 2C).

### Plasma proteome signatures in anti-PD-1 responders

**Figure 4:**
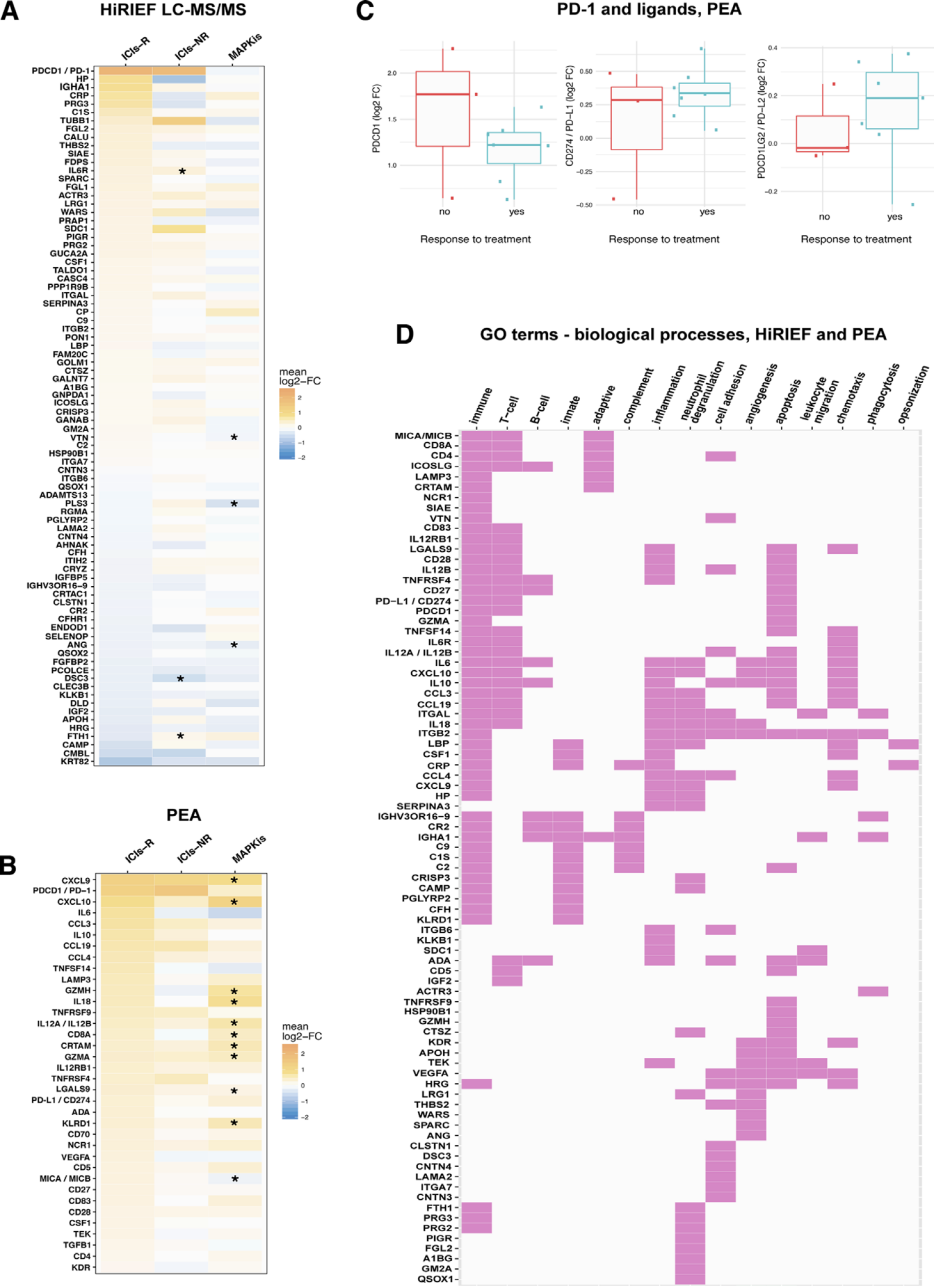
Stratification analyses on response to anti-PD-1 therapy in metastatic cutaneous melanoma. (A) HiRIEF LC-MS/MS heat map of the 84 proteins with a statistically significant change (log2-FC) in plasma protein levels during treatment in the subgroup of anti-PD-1-R, with corresponding changes during treatment in anti-PD-1-NR and patients treated with MAPKis. All comparisons were between trm and pre-trm matching plasma proteomes in the corresponding subgroup, using a paired t test at α=0.05. Some proteins that had a statistically significant log2-FC in plasma levels in anti-PD-1-R had a statistically significant change in anti-PD-1-NR and in patients treated with MAPKis (marked with an *). (B) Heat map on statistically significant change in protein plasma levels during treatment in anti-PD-1-R based on PEA data, with corresponding changes during treatment in anti-PD-1-NR and in patients treated with MAPKis. Although only a certain proportion of the proteins that had a statistically significant log2-FC in plasma levels in anti-PD-1-R had a statistically significant change in patients treated with MAPKis (here marked with a *), the size of the log2-FC indicates that some of the findings were not statistically significant due to the small sample sizes in the anti-PD-1-NR. This is supported by the observations in PD-1 log2-FC in anti-PD-1-R and anti-PD-1-NR (C). (D) Map of proteins with a change in plasma levels during treatment in anti-PD-1-R, which had an involvement in immune biological processes according to GO terms (HiRIEF LC-MS/MS and PEA data). Response to treatment included CR, PR, and SD. CR, complete response; HiRIEF LC-MS/MS, high-resolution isoelectric focusing liquid chromatography–mass spectrometry; GO, gene ontology; ICIs, immune checkpoint inhibitors; ICIs-NRs, patients who were treated with anti-PD-1 ICIs and had no response to treatment (anti-PD-1-NR); ICIs-R, patients who were treated with anti-PD-1 ICIs and responded to treatment (anti-PD-1-R); log2-FC, log2-fold-change; MAPKis, mitogen-activated protein kinase inhibitors; PD-1, programmed cell death protein 1; PEA, proximity extension assays; PR, partial response; pre-trm, pre-treatment; SD, stable disease; trm, after the first treatment cycle.

Stratifying the patients according to best response showed that 84 proteins had altered plasma levels during treatment in the subgroup of anti-PD-1-R in HiRIEF data (p<0.05, no FDR), with PD-1 showing the largest increase. Six of these proteins also showed a significant change during treatment in the MAPKi cohort or among the anti-PD-1-NRs (figure 4A; online supplementary tables S4 and S5, additional file 2). Comparing the protein log2-FC in anti-PD-1-R with anti-PD-1-NR showed differential alterations in plasma levels of 31 of the 1055 proteins quantified with HiRIEF LC-MS/MS (p<0.05, no FDR). Anti-PD-1-R had higher plasma levels of C1QC, LECT2, C1QA, C1QB, FCGR3A, B4GALT1, CFP, TPP1, LGALS3BP, proline-rich acidic protein 1 (PRAP1), and desmocollin 3 (DSC3), and lower plasma levels of IGHM, FTH1, CTGF, PLS3, LDHB, and LAMA2, as compared with anti-PD-1-NR (online supplementary figure S5, additional file 1; online supplementary table S6, additional file 2).

PEA analyses revealed 36 proteins with a significant change during treatment in anti-PD-1-R (p<0.05, 10% FDR), out of which 22 were specific for the anti-PD-1-R subgroup— among them PD-1, interleukin 6 (IL-6), and interleukin 10 (IL-10)— again recapitulating the significant change in PD-1 detected by the global analysis (figure 4B, online supplementary table S7 - additional file 2). PD-L1 again showed an increase in anti-PD-1-R during treatment (mean log2-FC=0.338, p=0.004, q=0.053). None of the proteins had a statistically significant change during treatment among anti-PD-1-NR in the PEA data. However, it is worth noticing that the number of anti-PD-1-NR is very small (n=3), making it less probable to detect changes of non-large effect size. Hence, changes in some of the proteins detected in anti-PD-1-R are still plausible in anti-PD-1-NR. A supporting observation in this regard is that of a high increase in plasma levels of PD-1 in two out of three NR patients during anti-PD-1 treatment (figure 4C). Due to the small sample size of anti-PD-1-NR in PEA data, none of the proteins had altered plasma levels during treatment in anti-PD-1-R when compared with anti-PD-1-NR.

To further confirm the role of the detected proteins in relation to response to treatment, we performed sensitivity analyses where we included only the four patients with CR (anti-PD-1-CR) as most representative of responders. Anti-PD-1-CR had the most evident clinical response to treatment and hence would show the most pronounced effects. In HiRIEF data, we detected a change during treatment in 44 proteins in anti-PD-1-CR (p<0.05, no FDR), of which 15 proteins were detected in the previous analysis of the entire subgroup of anti-PD-1-R. Again, PD-1 had the highest increase during treatment in anti-PD-1-CR (mean log2-FC=1.924, p=0.028). Other proteins with increased levels during treatment in anti-PD-1-CR were PRAP1, LRG1, A1BG, C9, and PPP1R9B, whereas IGHV3OR16-9 had the largest decrease (online supplementary figure S6a, additional file 1). The PEA sensitivity analysis detected a significant change in 10 proteins in anti-PD-1-CR during treatment (p<0.05, no FDR, online supplementary figure S6b), confirming an increase in PD-1 (mean log2-FC=1.112, p=0.007, q=0.170). Comparing the protein log2-FC in anti-PD-1-CR with anti-PD-1-NR showed differential alteration in plasma levels of 18 out of 1055 proteins quantified with HiRIEF LC-MS/MS (p<0.05, no FDR), recapitulating 11 proteins detected in the main analysis (online supplementary figure S7, additional file 1). Anti-PD-1-CR had higher levels of C1QB, LGALS3BP, PRAP1, MXRA5, DSC3, AHSP, and SSC5D, and lower levels of LAMA2, PLS3, LDHB, and GALNT6, as compared with anti-PD-1-NR.

Overall, the majority of the proteins detected to change in plasma during treatment in anti-PD-1-R were involved in T-cell response, inflammation, neutrophil degranulation, and cell adhesion, according to their corresponding GO terms on biological processes (figure 4D). Several acute phase proteins showed increased levels during treatment, among them C reactive protein (CRP), lipopolysaccharide binding protein (LBP), haptoglobin (HP), and IL-6, indicating a strong acute inflammation ongoing in anti-PD-1-R. An increase was also detected in plasma levels of cell adhesion proteins involved in building the interactions between the cells and the extracellular matrix (eg, SDC1, ITGB6, and ITGB2). In addition, 24 proteins associated with neutrophil degranulation were detected, correlating to reports that NLR can be a predictor of longer OS in patients with stage IV melanoma.^11 21^ Altogether, some of these findings coincide with previous reports on the involvement of immunological proteins in antitumor response to CM.^22–28^

### Protein plasma levels can predict PFS

**Figure 5:**
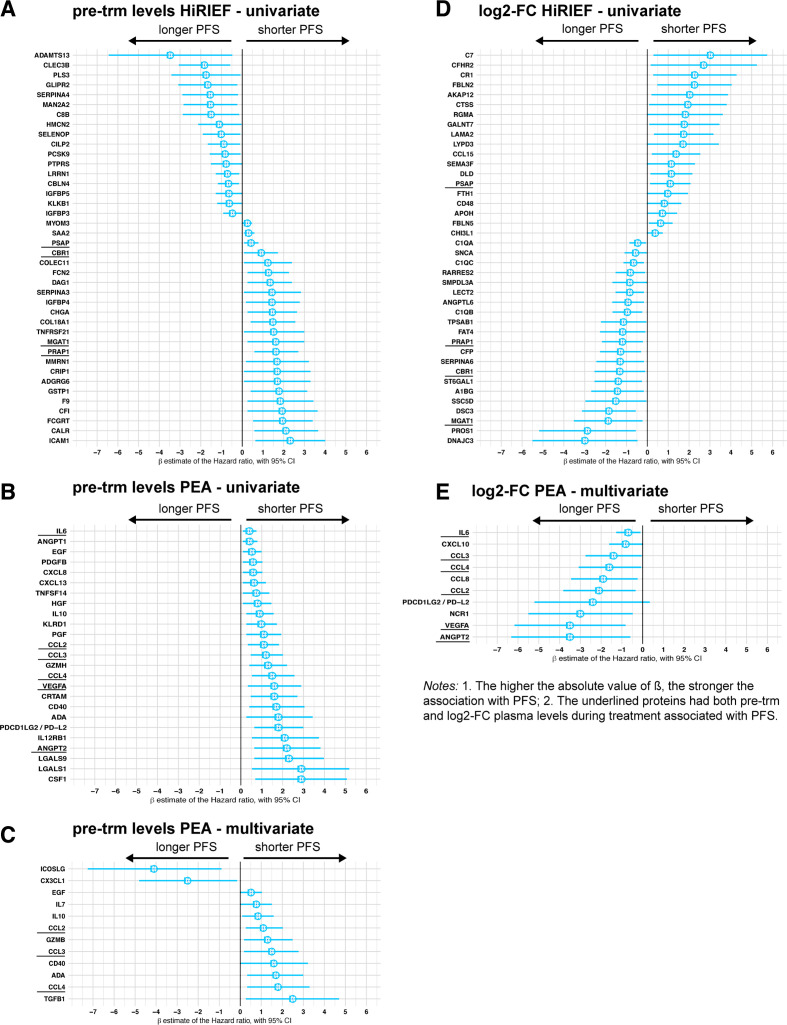
Forest plots on association between protein plasma levels and progression-free survival (PFS), Cox models. High-resolution isoelectric focusing liquid chromatography–mass spectrometry (HiRIEF LC-MS/MS) data, levels pre-trm (A) and log2-fold change (log2-FC) (D); Proximity extension assays (PEAs) data, levels pre-treatment (pre-trm) (univariate analyses—B, multivariate—C) and log2-fold change (E). Dots = β estimate=log2 of HR, interval bars=95% CI of the β estimate. The β in HiRIEF LC-MS/MS data is underestimated (see section on Cox models in Additional File 1). HiRIEF data included only patients with anti-programmed cell death protein 1 (anti-PD-1) treatment, whereas PEA data included patients treated with anti-PD-1 and/or anti-cytotoxic T-lymphocyte–associated antigen 4.

To detect potential predictive biomarkers, we also analyzed the association between pre-trm plasma protein levels and PFS. No clinical variables were associated with PFS in the HiRIEF LC-MS/MS-analyzed subgroup of anti-PD-1 treatment cohort (n=13); therefore we performed univariate analyses, which showed associations between protein pre-trm plasma levels of 109 proteins and PFS in this cohort (p<0.05, no FDR; online supplementary table S8, additional file 2). No overlap was detected between the anti-PD-1 and the MAPKi cohort in proteins with pre-trm levels associated with PFS. The proteins with the strongest association with PFS based on the HR estimate are plotted on figure 5A. Twenty-two proteins that had pre-trm levels associated with PFS in the univariate survival analyses remained significant after adjusting for age, sex, and LDH levels in sensitivity multivariate analyses (online supplementary table S9, additional file 2).

Due to the small sample size and insufficient power in the PEA analyses on PFS in the anti-PD-1 treatment cohort, patients who received anti-CTLA-4 treatment were also included as ICI patients (n=18). Association between higher plasma levels of 25 PEA proteins and shorter PFS in the ICI cohort was detected with univariate analyses of PEA data (p<0.05, 10% FDR; figure 5B). Eleven of the proteins also showed an association with PFS in the MAPKi cohort (online supplementary table S10, additional file 2). Age and sex were the only clinical variables that were significantly associated with PFS in the PEA analyses, and after adjusting for them 7 of the 25 proteins from the univariate analyses remained significant for predicting shorter PFS in the multivariate analyses as well (p<0.05, 10% FDR; figure 5C)—epidermal growth factor (EGF), IL-10, C-C motif chemokine ligands 2, 3 and 4 (CCL-2, -3, -4), CD40, and adenosine deaminase (ADA). After further adjustment for age, sex, and LDH levels, pre-trm levels of all the proteins that were associated with PFS in the main analysis (figure 5E) remained associated with PFS; furthermore, higher pre-trm levels of PD-L1 were associated with longer PFS (online supplementary table S11, additional file 2).

To detect potential early response biomarkers, we then compared the log2-FC in protein plasma levels and PFS. Univariate Cox models based on HiRIEF LC-MS/MS data showed that increase in 21 protein plasma levels was associated with longer PFS in anti-PD-1-treated patients, whereas an increase in plasma levels of 19 proteins was associated with shorter PFS (p<0.05, no FDR; figure 5D). Only one protein overlapped with the MAPKi cohort—synuclein alpha (SNCA). Interestingly, 9 of 40 proteins associated with PFS were also previously detected as altered in anti-PD-1-R during treatment: DSC3, A1BG, PRAP1, APOH, FTH1, DLD, LAMA2, GALNT7, and RGMA. Fifteen proteins that were associated with PFS in the univariate Cox models, for example, PRAP1, DSC3, C1QC, and LAMA2, remained significant after adjustment for age, sex, and LDH levels in sensitivity multivariate analyses (online supplementary table S12, additional file 2). Interesting enough, PD-1 log2-FC in HiRIEF data showed association with longer PFS.

After adjusting for age and sex, increase in plasma levels of nine proteins analyzed with PEA was associated with longer PFS in the ICI cohort (p<0.05, 10% FDR; figure 5E). Change in CCL2 during treatment was associated with longer PFS in both the ICI-treated and MAPKi-treated patients. Finally, CCL-2, CCL-3, CCL-4, IL-6, VEGFA, and angiopoietin 2 (ANGPT2), remained significant after further adjustment for age, sex, and abnormal LDH levels (online supplementary table S13, additional file 2). The association between PD-1 and PFS in PEA data was in the same direction as the PFS analyses in HiRIEF (protective), but not statistically significant.

### Detection of tumor-derived proteins carrying coding mutations

We and others have previously shown that by creating custom-made databases based on genomic sequence information, including genes containing coding mutations or splice variants, the corresponding altered proteins can be detected using HiRIEF LC-MS/MS-based proteogenomics.^14 15^ To explore if we could detect proteins containing coding sequence alterations that could potentially derive from the tumor, we searched again the HiRIEF LC-MS/MS data against a custom-made database (human VarDB^20^) that includes peptide sequences containing somatic mutations derived from the Catalogue of Somatic Mutations in Cancer (COSMIC).^14^

We detected in total 1197 coding variants in 381 genes covered by over 1200 peptides, including variants in PTEN, FGFR2, NRAS, and KIT (online supplementary dataset, additional file 3). Among the variant peptides with high levels in non-responders was a mutated peptide from the B2M protein, which has been previously linked to ICI therapy resistance in melanoma.^29^ This highlights how variant peptides could be used in a liquid biopsy approach to detect tumor-specific proteins in plasma.

10.1136/jitc-2019-000204.supp3 Supplementary data

Overall, the findings demonstrate the potential of HiRIEF LC-MS/MS as a liquid biopsy method, able to detect protein variants and a wide range of protein concentrations in plasma, including low-abundant proteins usually detectable only with targeted methods, such as PD-1.

## Discussion

To our knowledge, with a total of 1917 proteins identified and 1237 proteins analyzed, this is the first comprehensive study of the plasma proteome for patients with metastatic CM receiving ICIs, which provides a unique insight into the change in circulating proteins during treatment. Although with a limited sample size, the study design and the analytical approach allowed us to pinpoint molecular changes in plasma that were specific to anti-PD-1 therapy and to differentiate them from general treatment-induced changes by analyzing a cohort of patients with metastatic CM treated with MAPKis for comparison. MAPKis also induce immune modulation,^30^ which allowed us to differentiate generic immune processes that appear during treatment from specific immune processes stirred by ICIs. An example of a common process induced by both treatments is neutrophil degranulation, which was among the biological processes with higher proportion of proteins changing in both treatment cohorts.

The major finding of this study is the increase in circulating PD-1 in response to anti-PD-1 treatment, and in anti-PD-1-R. We used two principally different analytical techniques, HiRIEF-LC-MS/MS and PEA, and both showed the same increase in PD-1 plasma levels in response to anti-PD-1 treatment, which was not observed for the MAPKi cohort. In line with our findings, Music *et al* 31 have recently reported an increase in plasma levels of PD-1 in response to pembrolizumab in a mixed cancer cohort of 24 patients, among them 9 patients with CM.^31^ Interestingly, they report an increase in PD-1 7 weeks after treatment initiation, suggesting that the early elevation detected in our study remains later on during treatment.

The function and origin of soluble forms of PD-1 (sPD-1) and its ligands PD-L1/-L2 (sPD-L1/L2) have not been fully investigated.^32^ Early mRNA studies have suggested that the sPD-1 is a splice variant of the full-length membranous PD-1 protein lacking exon 3, which contains the transmembrane domain.^33^ Expression of both the full-length PD-1 and sPD-1 mRNAs has been reported in T cells,^33^ whereas in mice the extracellular domain of PD-1 has demonstrated potent enhancement of antigen-specific CD8+ T-cell responses elicited by vaccines.^34^ In line with these findings, one could speculate that coexpression of sPD-1 may enhance the effect of anti-PD-1 treatment by providing an endogenous PD-L1 inhibition in parallel with the drug-induced inhibition.

Another interesting aspect is whether the anti-PD-1 antibodies nivolumab and pembrolizumab have affinity toward the soluble version of PD-1 and accordingly if sPD-1 is bound to the drugs. Should the drugs have affinity to sPD-1, the increase in sPD-1 during treatment could be due to reduced clearance from the circulation, with the drug binding sPD-1 into an sPD-1-antibody complex, as observed in anti-tumor necrosis factor therapy.^35^ However, several observations from the current study suggest that sPD-1 is not bound to the anti-PD-1 antibodies. First, in the HiRIEF LC-MS/MS analysis, the initial step includes an antibody-based removal of 14 high-abundant proteins, including IgGs. The removal is performed in native conditions, which should remove any protein bound to IgG. As nivolumab and pembrolizumab are both IgG4 antibodies, they would have been removed at this stage together with any protein(s) bound to the antibody. An increase in sPD-1 levels during anti-PD-1 therapy would hence be less likely if the sPD-1 was bound to the drug. Moreover, in the PEA analyses two polyclonal antibodies toward PD-1 are used to detect the PD-1 protein. Again, if nivolumab or pembrolizumab were bound to sPD-1 they would likely block the epitopes for the PEA analysis and hence an increase in sPD-1 during treatment would be less likely to detect. Nonetheless, more studies are needed to decipher the exact role of sPD-1 in anti-PD-1 therapy.

In addition to PD-1, we also detected an increase in soluble PD-L1 (sPD-L1) during treatment in anti-PD-1-R by PEA analysis, although PD-L1 was not targeted by any of the drugs. sPD-L1 has been previously analyzed as a treatment predictive and prognostic biomarker in several cancers.^36–38^ Increased PD-L1 expression after anti-PD-1 treatment has been reported in matched post-treatment and pretreatment melanoma tissue samples,^39^ which suggests that the elevated sPD-L1 most likely derives from the tumor. Furthermore, we have detected an increase in plasma levels of several proteins involved in interferon (IFN) signaling, which could explain the increase in sPD-L1. IFN signaling increases the expression of PD-L1 in tumor tissue during ICI treatment^40^ and has been associated with predicting response to ICIs in some patients.^41^ Eventually, sPD-L1 might also be involved in suppressing the PD-1-mediated negative regulation of the T-cell response with an effect similar to that of sPD-1.^36 42^

We detected changes in several other proteins related to the T-cell activity in anti-PD-1-R and an increase in immune-suppressive proteins such as TGFB1, IL-10, and LGALS9, which may correlate to the dynamic interplay between the immune system and inflammatory tumor responses during anti-PD-1 treatment observed in tissue. Huang *et al* observed that although the immune system responds with a PD-1+ CD8+ T-cell infiltration and an inflammatory response after a single dose of anti-PD-1 ICIs, the tumor develops resistance mechanisms of immune suppression and tumor evolution in response to treatment.^39^ Furthermore, it is likely that the role of these molecules is complex and depending on the cell environment, as it is the case for IL-10, an established immunosuppressive protein that has been demonstrated to induce a strong antitumor T-cell response in mice and humans.^43 44^

Several of the proteins that were differentially altered (-up/-down) in plasma of anti-PD-1-R, as compared with anti-PD-1-NR were also predictive of PFS. Furthermore, several of these proteins remained consistently associated with PFS after adjusting for age, sex, and abnormal LDH levels in sensitivity multivariate analyses, for example, PRAP1, DSC3, C1QC, LAMA2, CCL2, CCL3, CCL4, IL-6, and VEGFA.

The PFS is a reliable treatment outcome that is directly linked to the treatment effect and less affected by subsequent treatment confounders that can affect OS. Analyzing the association with PFS can show the role of the plasma proteins as potential biomarkers and the biological processes in which they are involved, which favor or hinder response to treatment. Curiously, in the PFS survival analyses high pre-trm levels of a subset of inflammatory proteins were associated with shorter PFS for both the ICI and MAPKi cohort, whereas an increase in their levels during ICIs treatment was associated with a protective effect and longer PFS (ie, IL-6, CCL2, CCL3, CCL4, and VEGFA). This emphasizes the importance of timing in plasma sampling and how the temporal effects affect the role of proteins as biomarkers.

Last, in a proof-of-concept analysis, we also show that by employing a proteogenomics approach we can detect proteins harboring coding variants, similar to the liquid biopsy methods to detect cell free DNA, an approach that has shown to reflect the overall mutational profile of tumors as accurately as singe biopsies.^12^

## Conclusions

In this discovery study, we demonstrated increased levels of circulating PD-1 and PD-L1 in plasma of patients with metastatic CM during anti-PD-1 treatment, as well as diverse immune plasma proteomic signatures, which require validation in independent larger cohorts with targeted approaches. Moreover, we highlight the potential of combined, global, and targeted proteomics in discovering new associations between plasma proteins and anti-PD-1 treatment outcome in patients with metastatic CM and unraveling systemic biological processes during treatment. Diverse plasma proteomic signatures involved in different immune-related and tumor-derived processes are likely to provide a more reliable treatment prediction in CM, considering the molecular heterogeneity of the tumor and the systemic molecular processes attributable to ICI treatment.

10.1136/jitc-2019-000204.supp4 Supplementary data

## Data Availability

Data are available in a public, open access repository. Clinical data are available on reasonable request. All data relevant to the study are included in the article or uploaded as supplementary information. All data generated or analyzed during this study are included in this published article and the supporting information in Additional File 1. Mass spectrometry raw data have been uploaded to the PRIDE repository through the ProteomeXchange, with accession number PXD017201. Data from proteogenomic analyses are available in a supplementary dataset in Additional File 3, and data from the PEA analyses are available in a supplementary dataset in Additional File 4.
