# Supplementary material for: In-depth plasma proteomics reveals increase in circulating PD-1 during anti-PD-1 immunotherapy in patients with metastatic cutaneous melanoma
Source: J Immunother Cancer. 2020 May 20;8(1):e000204. doi: 10.1136/jitc-2019-000204 (PMC7253007; doi:10.1136/jitc-2019-000204)
Supplement: Supplementary data [file jitc-2019-000204supp001.pdf]

Additional File 1

Supplementary methods and figures for study:

In-depth plasma proteomics reveals increase in circulating PD-1 during anti-PD-1 immunotherapy in patients with metastatic cutaneous melanoma. Haris Babačić, Janne Lehtiö, Yago Pico de Coaña, Maria Pernemalm, Hanna Eriksson. 2020

Supplementary information on Methods

Patient characteristics and plasma samples

The blood samples were obtained between March 2013 and August 2016, at the Department of Oncology, Karolinska University Hospital, Sweden. Blood was collected in EDTA tubes before and during treatment from melanoma patients with disseminated disease undergoing first line therapy with ICIs or MAPKis.

The blood was centrifuged at 1,500 g for 10 minutes and then the separated plasma was centrifuged 2,400 × g for 15 minutes. All samples were stored at −70°C until they were assayed.

The M1c and M1d patients were grouped together because of the small number of M1d patients (n = 7). LDH values > 3.5 mKat/L were considered elevated for the age group 18-70 years and > 4.3 mKat/L for the age group >70 years according to the upper reference levels at the Karolinska University Laboratory.

An SD-PR response or “mixed response” (i.e. increased size of some lesions and decreased size of others) were confirmed by imaging performed within 6-8 weeks.

HiRIEF LC-MS/MS

High abundant protein depletion and in-solution digestion

The depleted plasma flow-through was concentrated on 5 kD molecular weight cut off filter (Agilent Technologies), followed by buffer exchange >100 times to 25mM HEPES, pH 7.6.

In-solution digestions with LysC and trypsin. Briefly, depleted plasma was denatured at 95°C for 5 minutes followed by reduction with dithiothreitol and alkylation with iodoacetamide at end concentrations of 5mM and 10mM respectively. LysC was added at a 1:50 (w/w) ratio and digestion was performed at 37°C overnight. The samples were further digested by trypsin at a 1:50 (w/w) ratio with 37°C overnight incubation. After LysC/trypsin digestion, ~1% of each peptide sample was aliquoted for ~15min gradient liquid chromatography-mass spectrometry (LC-MS/MS) runs to check for protease activity by the samples' miscleavage rate.

LC-MS/MS analysis

Online LC-MS was performed using a Dionex UltiMate™ 3000 RSLCnano System coupled to a Q-Exactive mass spectrometer (Thermo Scientific). Each plate well was dissolved in 20ul solvent A and 10ul were injected. Samples were trapped on a C18 guard-desalting column (Acclaim PepMap 100, 75μm x 2 cm, nanoViper, C18, 5 µm, 100Å), and separated on a 50cm long C18 column (Easy spray PepMap RSLC, C18, 2 µm, 100Å, 75 µm x 50 cm). The nanocapillary solvent A was 95% water, 5%DMSO, 0.1% formic acid; and solvent B was 5% water, 5% DMSO, 95% acetonitrile, 0.1% formic acid. At a constant flow of 0.25 µl min^{-1}, the curved gradient went from 2% B up to 40% B in each fraction as shown in the supplementary table 1, followed by a steep increase to 100% B in 5 min and subsequent re-equilibration with 2% B.

FTMS master scans with 70,000 resolution (and mass range 300-1700 m/z) were followed by data-dependent MS/MS (35 000 resolution) on the top 5 ions using higher energy collision dissociation (HCD) at 30% normalized collision energy. Precursors were isolated with a 2 m/z window. Automatic gain control (AGC) targets

were 1e6 for MS1 and 1e5 for MS2, with minimum AGC target of 1e3. Maximum injection times were 100 ms for MS1 and 450 ms for MS2. The entire duty cycle lasted ~2.5 s. Dynamic exclusion was used with 30.0s duration. Precursors with unassigned charge state or charge state 1, 7, 8, >8 were excluded.

Data searches

Raw MS/MS files were converted to mzML format using msconvert from the ProteoWizard tool suite (1). Spectra were then searched in the Galaxy framework using tools from the Galaxy-P project (2,3), including MSGF+ (4) (v10072) and Percolator (5) (v2.10), where 8 subsequent HiRIEF search result fractions were grouped for Percolator target/decoy analysis. Peptide and PSM FDR were recalculated after merging the percolator groups of 8 search results into one result per TMT set. The reference database used was the human protein subset of ENSEMBL 80. Quantification of isobaric reporter ions was done using OpenMS project's IsobaricAnalyzer (6) (v2.0). Quantification on reporter ions in MS2 was for both protein and peptide level quantification based on median of PSM ratios, limited to PSMs mapping only to one protein and with an FDR q-value < 0.01. FDR for protein level identities was calculated using the -log10 of best-peptide q-value as a score. The search settings included enzymatic cleavage of proteins to peptides using trypsin limited to fully tryptic peptides. TMT6 on lysine and peptide N-terminus and Carbamidomethylation of cysteine were specified as fixed modifications. The minimum peptide length was specified to be 6 amino acids. Variable modification was oxidation of methionine.

Baba Ö‘, *et al. J Immunother Cancer* 2021; 8:e000204. doi: 10.1136/jitc-2019-000204

Proteogenomics search pipeline / SpectrumAI

The human VarDB consists of peptides originating from previously annotated nsSNPs, somatic mutations, pseudogenes, and long non-coding RNAs, forming a supplementary set of peptides to the canonical proteome.

Proximity extension assays (PEA)

Measurements were performed using 1 µL of each sample. In PEAs, a pair of oligonucleotide-labelled antibodies binds to their targeted protein in the samples. If the probes are in close proximity the oligonucleotides will hybridize in a pair-wise manner. The addition of a DNA polymerase leads to a proximity-dependent DNA polymerization event, generating a unique PCR target sequence. The target sequence is detected and quantified using a microfluidic real-time PCR instrument (Biomark HD, Fluidigm). Data is then quality controlled and normalised using an internal extension control and an inter-plate control, to adjust for intra- and inter-run variation.

Statistical analysis

Baseline characteristics comparison of response strata

We analysed the baseline characteristics between anti-PD-1-R and anti-PD-1-NR with a two-sided *t* test, *Wilcoxon* test, or *Fisher* test, corresponding to the data characteristics, at $\alpha = 0.05$, and PFS and OS with Kaplan-Meier (K-M) curves and a two-sided *log-rank* test, at $\alpha = 0.05$.

Agreement analysis

We compared the agreement between the LC-MS/MS and PEA measurements, using ordinary least products (model II) linear regression, on

quantifying plasma levels and the change during treatment of proteins that were detectable with both methods and were analysed in the same plasma samples, in order to estimate the measurement bias in the two methods.

Multiple testing

In all the analyses, we used the Benjamini-Hochberg procedure to correct for multiple testing and adjust the *p* values. Initially, we have decided to use FDR threshold of $q = 0.1$ in all the analyses. However, because of the distribution of high *q*-values (between 0.3-1) obtained after FDR correction in the HiRIEF LC-MS/MS data and the discovery nature of this study, we have decided to base our interpretation on the *p* values, in order to prevent type II error (false negatives). This was evident in the case of PD-1, which we have validated with another method. Matching of patients allowed the use of a paired *t* test, which has a more stable type I error than the independent *t* test. We calculated Cohen's d for proteins with a significant change during treatment, in order to differentiate the clinical relevance of the discovered change. Proteins with larger effect sizes of the change, as estimated with Cohen's *d*, are findings with greater confidence. Due to the small sample size, the effect size estimates are likely overestimated.

Distribution of GO terms

We described the distribution of the proteins' GO terms on their molecular function, involvement in biological process, and their origin as a cellular component, in order to get an overview of ontologies of the proteins that changed in patients' plasma during treatment. The GO terms' distribution plot shows the most prevalent biological processes according to the GO terms of the proteins that had a differential alteration in plasma levels during treatment.

Stratification sensitivity analysis

The patients with SD who were included in the stratum of responders (anti-PD-1-R) were followed and alive for at least two years after treatment start and hence it is justifiable to include them in the stratum of anti-PD-1-R. Nonetheless, patients with stable disease (SD) can sometimes be difficult to categorise, which can contribute to uncertainty in categorisation of best response. In order to assess the uncertainty of the stratification and how it affected the differential analysis in the anti-PD-1 responders (anti-PD-1-R) stratum by including patients with PR and SD, we performed a sensitivity analysis where we included only patients with complete response (CR) as responders. Patients with CR (anti-PD-1-CR) have a clinically most evident response and are most representative of responders. In the first sensitivity analysis we compared trm to pre-trm levels in anti-PD-1-CR, and in the second sensitivity analysis, we compared the log2-fold change (log2(trm) – log2(pre-trm)) in anti-PD-1-CR to anti-PD-1 non-responders.

With the sensitivity analyses we have addressed whether the inclusion of patients with PR and SD in the stratum of responders has affected the main findings. This addresses the uncertainty of the analysis by excluding patients who might have affected the results. Where the findings from the main analyses remain consistent with the findings of the sensitivity analysis, one can be more confident that the inclusion of PR and SD did not significantly affect the main analysis.

Cox proportional hazards models

The following clinical variables were tested for association with PFS: age at baseline, sex, LDH levels, M stage, and response vs. no response to treatment, using a backward stepwise approach, and a two-sided likelihood ratio test (LRT), at $\alpha = 0.05$.

Baba Ö‘, H *et al. J Immunother Cancer* 2021; 8:e000204. doi: 10.1136/jitc-2019-000204

We adjusted for the clinical variables if they had an association with PFS in the analysed subgroup. If none of the variables was associated with PFS, we performed univariate analyses.

In HiRIEF data, we have performed the analyses only for the anti-PD-1 ICIs cohort. Due to the small number of PEA analytes in patients treated with anti-PD-1 ICIs (n = 10), we could not infer the association with PFS. Only for analysing proteins' associations with PFS in PEA-analysed plasma samples of ICIs patients, we included also patients treated with anti-CTLA-4 and both anti-CTLA-4 and anti-PD-1 ICIs.

In the HiRIEF LC-MS/MS the β estimates of the hazard ratios (HR) had extremely high and low values, probably because of the small sample size, which makes the HR overestimated. We transformed the data by multiplying the log2 values by 4, to reduce the values of the β estimates and shrink the variability, narrowing down the confidence intervals of the HR and penalising the value of the HR. The transformation did not affect the direction of the association of the change in protein levels during treatment with PFS or the statistical-significance of the β estimates. In the manuscript, we report the transformed β estimates. The reader should note that the reported β estimates and the width of the 95% CI are underestimated.

To address uncertainty, we have further arbitrarily adjusted the HR estimates in the Cox models for age, sex, and LDH levels (categorical: > upper limit of normal vs normal) in a sensitivity multivariate analysis on protein plasma levels (pre-trm or log2-FC) and PFS, in both HiRIEF LC-MS/MS data and PEA data.

We have not additionally analysed overall survival in this cohort of patients because some of the patients who had progression after an initial treatment with ICIs or MAPKis received additional treatments. These additional treatments would have been a confounder that would have affected the overall survival estimates. This is

most evident in Figure 1b, where the longest survivor is patient 35 who had initially been treated with MAPKis, had a progression, then received ICIs treatment and was still alive at the end of the study.

R packages used:

R packages for data handling: dplyr, plyr, oce, reshape2, gridExtra.

R packages for missing values: dataExplorer, Amelia.

R packages for visualisation: ggplot2, ggrepel, ggsignif, VennDiagram.

R packages for model 2 linear regression: lmodel2.

R packages for GO terms: biomaRt.

R packages for survival analysis: survival, survminer.

References for methods:

1. Kessner D, Chambers M, Burke R, Agus D, Mallick P. ProteoWizard: open source software for rapid proteomics tools development. Bioinformatics. England; 2008;24:2534–6.
2. Boekel J, Chilton JM, Cooke IR, Horvatovich PL, Jagtap PD, Kall L, et al. Multi-omic data analysis using Galaxy. Nat Biotechnol. United States; 2015;33:137–9.
3. Goecks J, Nekrutenko A, Taylor J. Galaxy: a comprehensive approach for supporting accessible, reproducible, and transparent computational research in the life sciences. Genome Biol. England; 2010;11:R86.
4. Kim S, Pevzner PA. MS-GF+ makes progress towards a universal database

search tool for proteomics. Nat Commun. England; 2014;5:5277.

5. Kall L, Canterbury JD, Weston J, Noble WS, MacCoss MJ. Semi-supervised learning for peptide identification from shotgun proteomics datasets. Nat Methods. United States; 2007;4:923–5.

6. Rost HL, Sachsenberg T, Aiche S, Bielow C, Weisser H, Aicheler F, et al. OpenMS: a flexible open-source software platform for mass spectrometry data analysis. Nat Methods. United States; 2016;13:741–8.

Supplementary Figures

a.

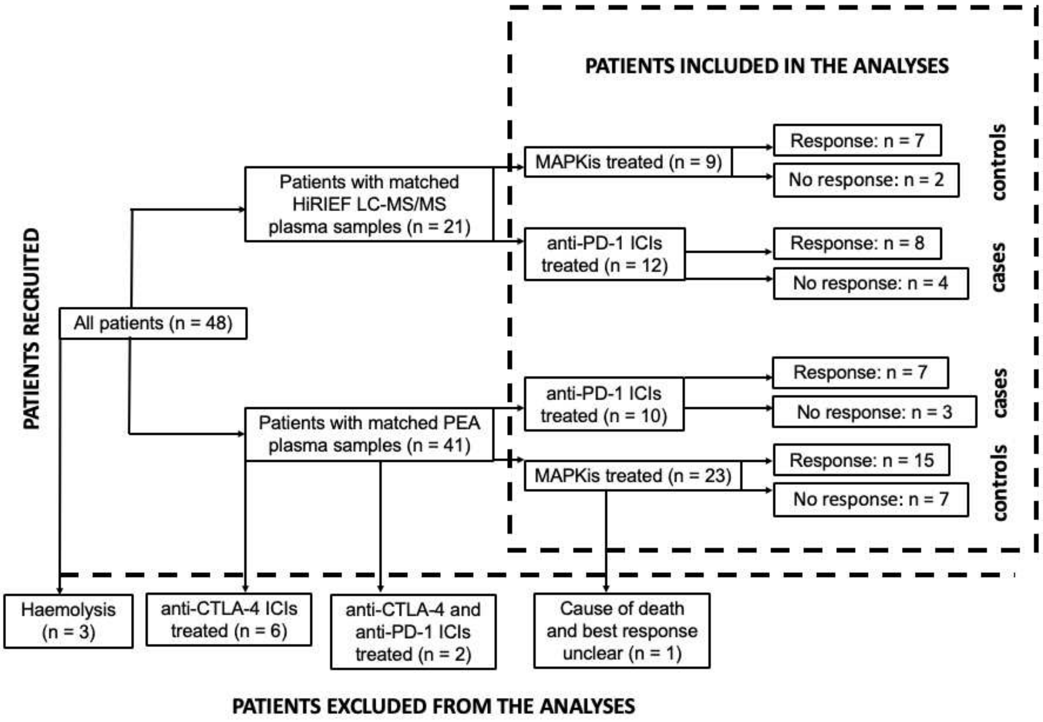

b.

Treatment characteristics	ICIs	MAPKis
Type of inhibitors – no.		
anti-PD1	12	0
anti-BRAF and anti-MEK	0	9
Treatment response – no.		
Complete response (CR)	4	1
Partial response (PR)	1	4
Stable disease (SD)	3	2
Non-responder (NR)	4	2

c.

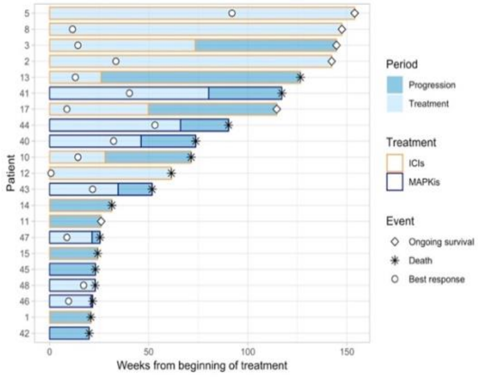

Figure S1. Characteristics of patients included in the analyses. a. Flowchart of patients' recruitment and stratification; **b.** Treatment and best response in patients analysed with HiRIEF LC-MS/MS; **c.** Swimmers' plot on patients with matched plasma samples analysed with HiRIEF LC-MS/MS;

a.

TMT label	Samples Pool 1	Sample taken	Samples Pool 2	Sample taken	Samples Pool 3	Sample taken	Samples Pool 4	Sample taken	Samples Pool 5	Sample taken
126	001	Pre-trm	010	Pre-trm	019	Trm	028	Pre-trm	037	Trm
127N	002	Trm	011	Trm	020	Pre-trm	029	Trm	038	Pre-trm
127C	003	Pre-trm	012	Pre-trm	021	Trm	030	Pre-trm	039	Trm
128N	004	Trm	013	Trm	022	Pre-trm	031	Trm	040	Pre-trm
128C	005	Pre-trm	014	Pre-trm	023	Trm	032	Pre-trm	041	Trm
129N	006	Trm	015	Pre-trm	024	Pre-trm	033	Trm	042	Pre-trm
129C	007	Pre-trm	016	Trm	025	Trm	034	Pre-trm	043	Trm
130N	008	Trm	017	Pre-trm	026	Pre-trm	035	Trm	044	Pre-trm
130C	009	Trm	018	Trm	027	Trm	036	Pre-trm	045	Trm
131	IS1	n.a.	IS2	n.a.	IS3	n.a.	IS4		IS5	

b.

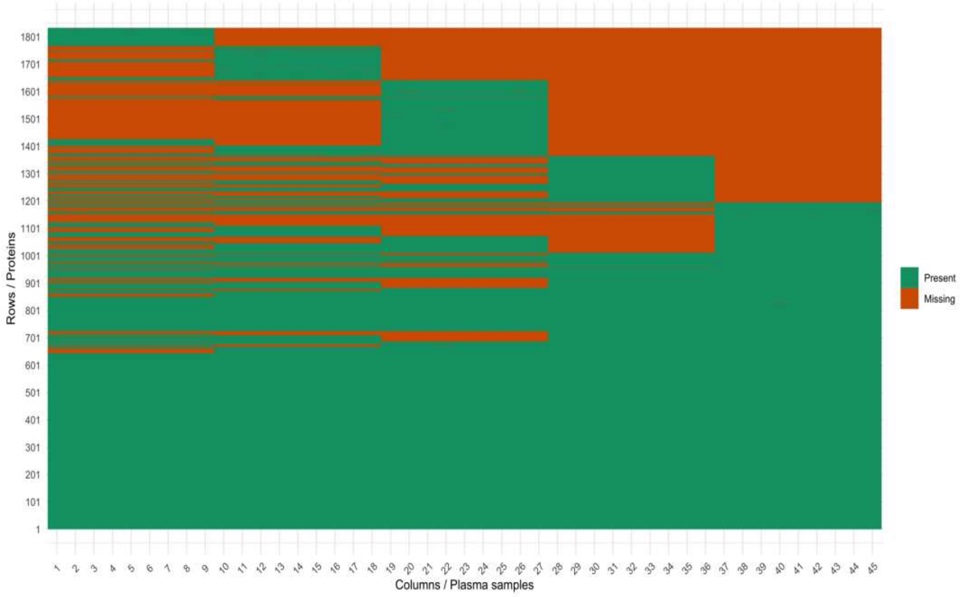

Figure S2. Batch effect of the TMT-pooling on the distribution of missing values.
a. TMT-labelling strategy in the HiRIEF LC-MS/MS analysis. The plasma samples labelled with “pre-trm” are taken before treatment, whereas those labelled with “trm” are taken during treatment. Samples labelled with blue are from patients treated with MAPKis, and samples labelled with orange are from patients treated with ICIs. The samples labelled with “IS” are internal reference standards.
b. Missingness map of the LC-MS/MS dataset with protein levels expressed as log2 values in the rows and patients’ samples in the columns. Samples from the same pool have almost the same distribution of proteins with missing values.

Abbreviations: ICIs – immune checkpoint inhibitors; MAPKis – MAPK-inhibitors;

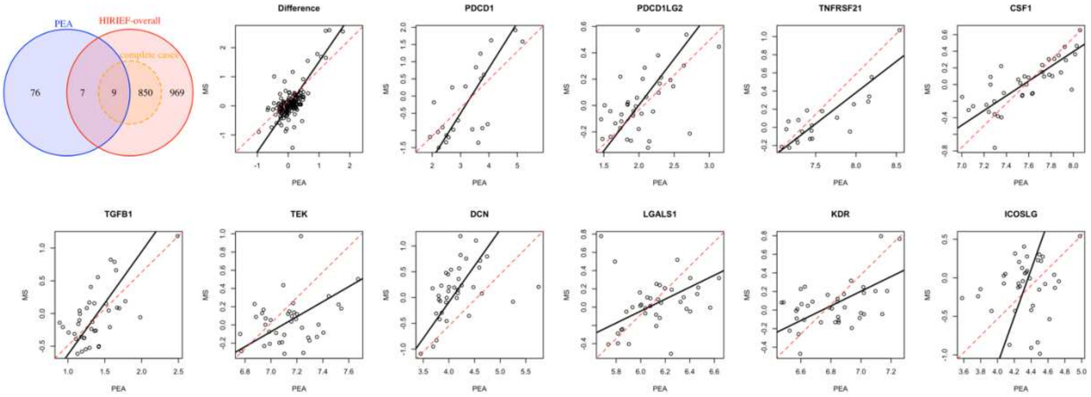

Figure S3. Agreement between HiRIEF LC-MS/MS and PEA quantification. The Venn diagram shows the overlap between HIRIEF-LC-MS/MS and PEA in protein detection. The agreement between the two methods is depicted with scatter plots on the levels of 10 proteins detected with both methods in >50% of observations for each method, as well as the change in protein levels during treatment (difference).

HiRIEF LC-MS/MS detected sixteen of the 92 PEA proteins in an unbiased manner. Ten proteins had observations in more than 50% of the samples for each method. Overall, the two methods showed varying agreement, with varying levels of fixed and proportional measurement bias. The highest agreement between HiRIEF LC-MS/MS and PEA was observed in the assessment of change in protein levels during treatment and quantifying the levels of PD-1, PD-L2, TGFB1, CSF1, and TNFRSF21.

Note: The full lines are ordinary least products linear regression lines on the relationship between the two methods. The dashed 45° lines represent imaginary linear relationships where there is no bias in quantifying proteins by the two methods. The disagreement between the two methods is represented by how much the regression lines deviate from the 45° lines. The deviation in the intercept of the regression lines represents fixed bias, whereas the deviation in the slope represents proportional bias.

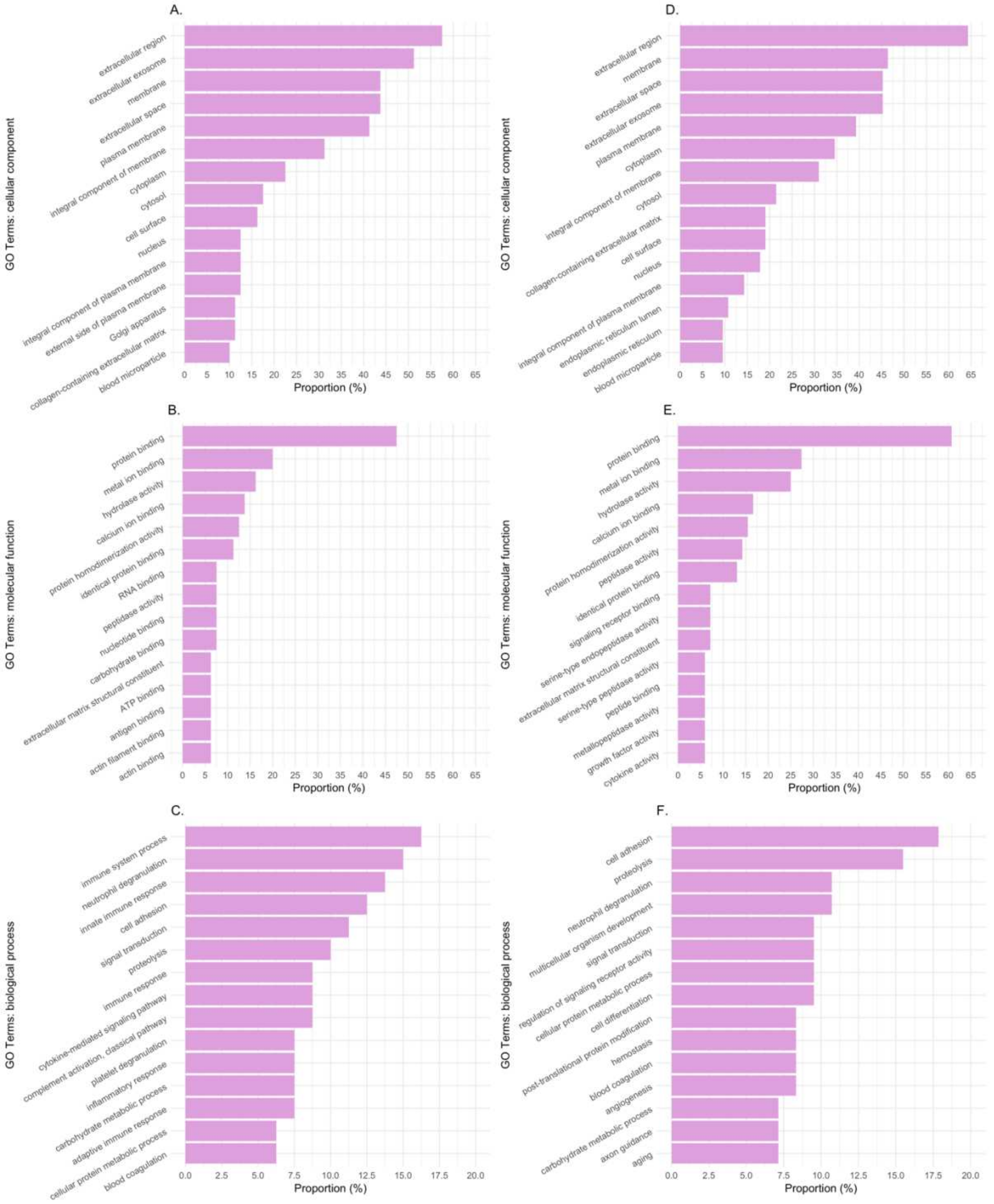

Figure S4. Classification of HiRIEF LC-MS/MS-detected proteins per GO terms in the two cohorts. The graphs show the most frequent GO terms associated with the proteins with a statistically-significant change in plasma during treatment with ICIs (A-C) or MAPKis (D-F).

Abbreviations: ICIs – immune checkpoint inhibitors; MAPKis – MAPK-inhibitors;

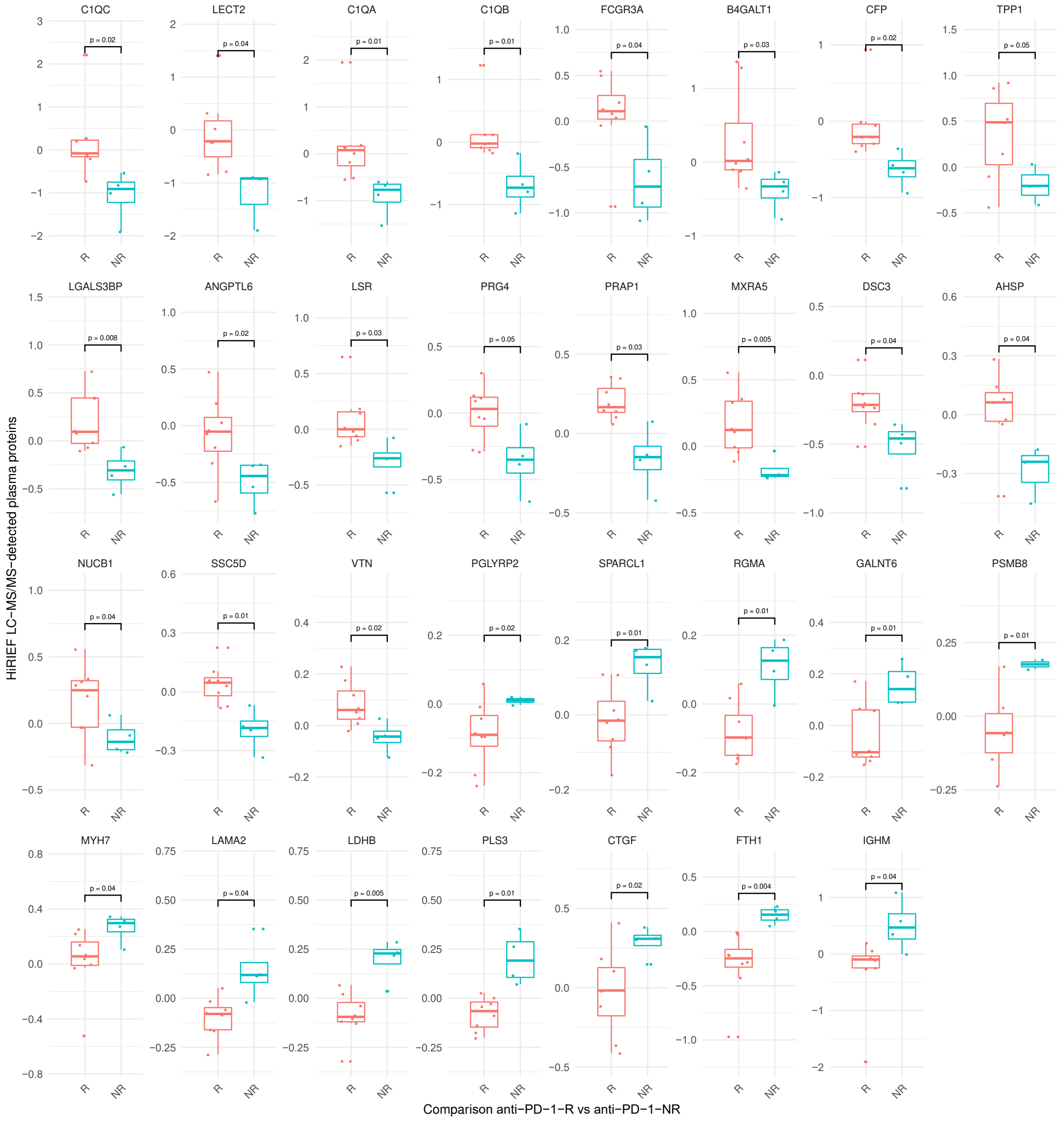

Figure S5. Proteins with differentially altered plasma levels in the anti-PD-1 immune checkpoint inhibitor subgroup of responders as compared to the subgroup of anti-PD-1 non-responders, HiRIEF LC-MS/MS. Values of log2-FC > 0 = higher plasma levels in responders, whereas log2-FC < 0 = lower levels in responders. The p values are obtained with a two-sided unpaired *t* test comparing the log2-FC in anti-PD-1 responders (R) vs anti-PD-1 non-responders (NR).
Patients who had complete response, partial response, or stable disease as best response during anti-PD-1 treatment were considered as responders, and patients who had progression were non-responders.

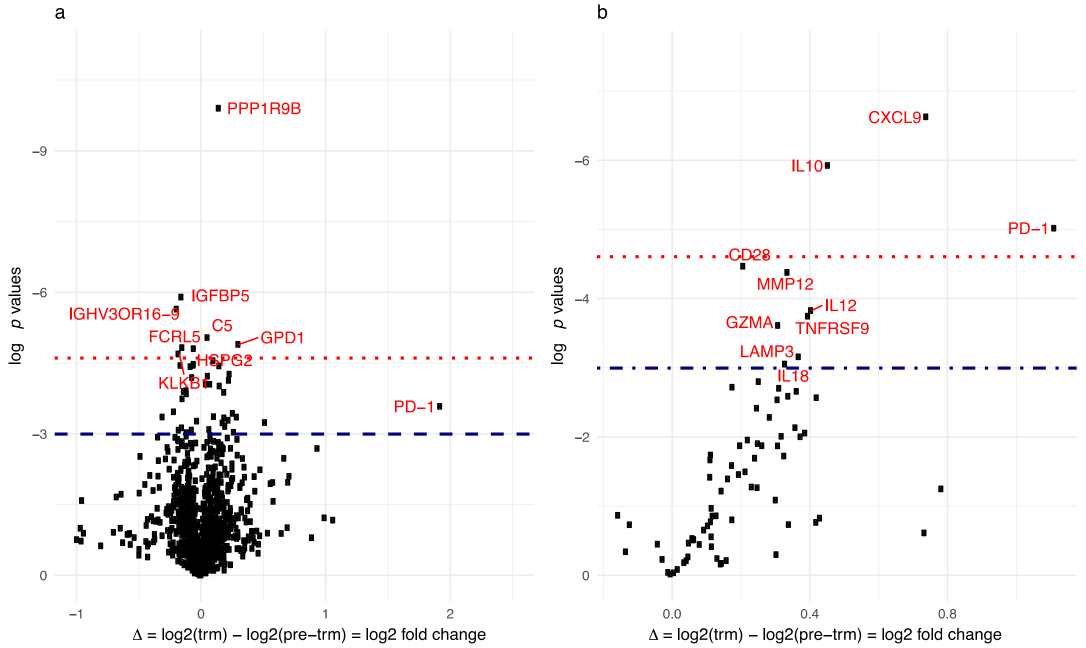

Figure S6. Sensitivity analysis on the change (log2-FC) in protein plasma levels during treatment in anti-PD-1 complete responders (anti-PD-1-CR). a. Volcano plots on comparing trm to pre-trm protein plasma levels in anti-PD-1-CR, HiRIEF LC-MS/MS analyses (paired *t* test, two-sided).
b. Volcano plots on comparing trm to pre-trm protein plasma levels in anti-PD-1-CR, PEA analyses (paired *t* test, two-sided).

Note: Proteins above the dashed line had $p < 0.05$, proteins in red and above the dotted line had $p < 0.01$.

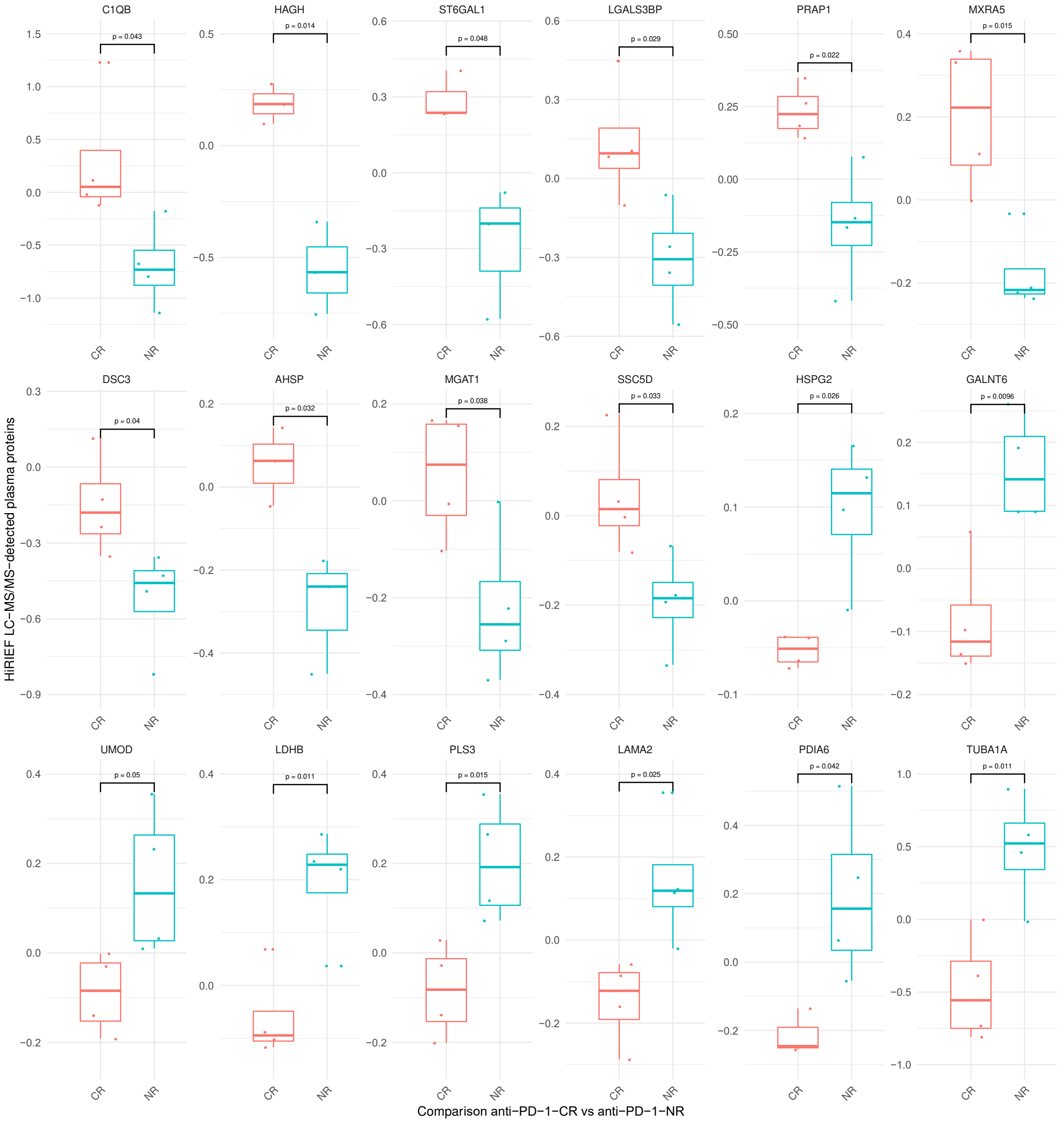

Figure S7. Proteins with differentially altered plasma levels in the anti-PD-1 immune checkpoint inhibitor subgroup of complete responders as compared to the subgroup of anti-PD-1 non-responders, HiRIEF LC-MS/MS. Values of log2-FC > 0 = higher plasma levels in complete responders, whereas log2-FC < 0 = lower levels in complete responders. The *p* values are obtained with a two-sided unpaired *t* test comparing log2-FC in anti-PD-1 complete responders (CR) vs anti-PD-1 non-responders (NR).
